# Impact of FcγR variants on the response to alemtuzumab in multiple sclerosis

**DOI:** 10.1002/acn3.50935

**Published:** 2019-11-04

**Authors:** Christian W. Keller, Tobias Ruck, Donal McHugh, Steffen Pfeuffer, Catharina C. Gross, Catharina Korsukewitz, Nico Melzer, Luisa Klotz, Sven G. Meuth, Christian Münz, Falk Nimmerjahn, Heinz Wiendl, Jan D. Lünemann

**Affiliations:** ^1^ Department of Neurology with Institute of Translational Neurology University Hospital Münster Münster Germany; ^2^ Laboratory of Viral Immunobiology Institute of Experimental Immunology University of Zurich Zurich Switzerland; ^3^ Department of Biology Institute of Genetics University of Erlangen‐Nürnberg Erlangen Germany

## Abstract

Allelic variants of genes encoding for the Fc gamma receptors IIIA and IIA have been associated with the clinical response to cell‐depleting antibodies in lymphoma patients. Here, we tested the hypothesis that *FCGR3A* and *FCGR2A* high‐affinity polymorphisms predict clinical outcomes to alemtuzumab therapy in 85 patients with relapsing‐remitting multiple sclerosis. No differences in clinical and MRI‐based efficacy parameters, the development of severe infusion‐associated reactions and secondary autoimmune diseases during a 2 year follow‐up was observed based on *FCGR3A* or *FCGR2A* polymorphisms. This study does not support the use of *FCGR* genetic variants to predict clinical outcomes to alemtuzumab.

## Introduction

Alemtuzumab (Sanofi Genzyme, Cambridge, MA) is a lymphocyte‐depleting humanized monoclonal IgG1 kappa antibody that selectively targets CD52, an antigen highly expressed on T and B cells. It is currently approved for highly active relapsing‐remitting multiple sclerosis (RR‐MS) in over 50 countries, and data from initial treatment cohorts as well as extension studies of phase III clinical trials provided evidence that alemtuzumab can induce prolonged disease remission with no evidence for disease activity in more than 50% of treated patients.^1^, ^2^ While the most common adverse events of alemtuzumab therapy are mild and moderate infusion‐associated reactions (IARs), a major concern with its use is the high risk of developing secondary autoimmune diseases (SAD) which peak around year 3 after treatment initiation with thyroid autoimmune disease (30–40% of patients) and immune thrombocytopenia (1–3% of patients) being the most frequent conditions.^1^, ^2^ Identification of biomarkers that can predict the clinical efficacy of alemtuzumab or the occurrence of adverse events would be of great value for the management of patients with MS.

Lymphocyte‐depleting antibodies such as alemtuzumab lyse target cells through IgG Fc‐mediated effector functions such as antibody‐dependent cell‐mediated cytotoxicity (ADCC), antibody‐dependent cellular phagocytosis (ADCP), complement‐dependent cytotoxicity (CDC), and by induction of apoptosis. ADCC and ADCP require engagement and cross‐linking of activating FcγR expressed by immune effector cells. In preclinical models, FcγR‐mediated cytotoxicity contributes substantially to the efficacy of depleting antibodies such as the anti‐HER2 antibody trastuzumab, rituximab, and alemtuzumab,^3^, ^4^ and higher affinity allotypic forms of the human activating FcγRIIIA as well as genotypic variants of the activating FcγRIIA were shown to be associated with improved clinical outcomes in rituximab‐treated lymphoma patients in some 5, 6, 7 but not all 8 studies.

The clinical efficacy of alemtuzumab to induce durable disease remission in MS is believed to involve the initial elimination of pathogenic lymphocytes.^9^ While peripheral blood leukocytes are usually efficiently depleted by alemtuzumab, FcγR binding affinities of alemtuzumab might additionally determine its efficacy to eliminate pathogenic T and B cells that are less accessible and reside at sites of inflammation such as lymphoid tissues or the central nervous system.^10^ Expression of the high‐affinity V allele with a valine at amino acid position 158 of *FCGR3A* results in tighter binding of the FcγRIIIA to IgG1 and IgG3, whereas the low‐affinity F allele with phenylalanine at this position is associated with decreased binding to IgG, resulting in less efficient ADCC.^11^, ^12^ Similarly, the high‐affinity H allele with histidine at amino acid position 131 of *FCGR2A* results in greater affinity of FcγRIIA for IgG2, whereas the low‐affinity R allele correlates with decreased IgG binding. Here we investigated whether FcγRIIIA and FcγRIIA polymorphisms are associated with clinical outcomes to alemtuzumab treatment in patients with MS.

## Subjects and Methods

### Patients and biomaterial

All patients were recruited in the Department of Neurology at the University Hospital Münster, Germany. 85 patients with RR‐MS on alemtuzumab treatment (Table 1) were included in the current study. For some patients not all parameters investigated were available which resulted in analyses of *n* = 85 – x for some characteristics. These dropouts included: NEDA‐3, one patient (F/F group); MRI stability, two patients (F/F and V/V groups) for FcγRIIIA and NEDA‐3, one patient (R/R group); MRI stability, two patients (H/R and R/R groups) for FcγRIIA. PBMCs were isolated from ethylenediaminetetraacetic acid blood derived from these patients during blood draws between 2014 – 2017 and cryopreserved as previously described.^13^

**Table 1:** Demographic characteristics of RR‐MS Patients (*n* = 85).

Female	49 (57.6%)
Age (mean, median, SD)	35.78, 35, 9.66
Age range (years)	18–58
Treatment naïve	15 (17.6%)
Prior treatment	70 (82.4%)
Patients with ≥ 2 relapses in the 2 years prior to 1^st^ infusion	60 (70.6%)
EDSS at baseline (mean, median, SD)	2.8, 2.5, 1.5
Ethnicity: Caucasian	85 (100%)

### Standard protocol approvals, registrations, and patient consents

This study was performed according to the Declaration of Helsinki and approved by the local ethics committee (2014‐398‐f‐S). All patients gave written informed consent.

### DNA isolation

DNA was isolated using Qiagen’s *DNeasy Blood & Tissue Kit* (Cat No.: 69506) according to the manufacturer’s recommendation.

### PCR testing and optimization

PCR primers were designed to amplify the region of the two SNPs of interest in *FCGR2A* (NP_067674.2 position 166) and *FCGR3A* (NP_000560.5, position 212). Due to high homology of *FCGR3A*, primers were designed in a region allowing the specific amplification of the *FCGR3A* gene. The PCRs were tested on a gradient and the optimal annealing temperature was chosen.

The analytical procedure was tested using six blood samples derived from donors with known *FCGR2A* and *FCGR3A* genotypes recruited at the University Hospital Erlangen, Germany. The results of the test runs are shown in Table 2.

**Table 2:** PCR design.

Sample	Sequence read	RNA Accession	Protein Accession	Nucleotide Position	Reference Nucleotide	Mutation Nucleotide	Amino Acid Position	Reference Amino Acid	Mutation Amino Acid
FcgRIIA‐131 H/H	for	NM_021642.3	NP_067674.2	c.497	A		166	H	
	rev	NM_021642.3	NP_067674.2	c.497	A		166	H	
FcgRIIA‐131 H/R	for	NM_021642.3	NP_067674.2	c.497	A	AG	166	H	H/R
	rev	NM_021642.3	NP_067674.2	c.497	A	AG	166	H	H/R
FcgRIIA‐131 R/R	for	NM_021642.3	NP_067674.2	c.497	A	G	166	H	R
	rev	NM_021642.3	NP_067674.2	c.497	A	G	166	H	R
FcgRIIIA‐158 F/F	for	NM_000569.6	NP_000560.5	c.634	T		212	F	
	rev	NM_000569.6	NP_000560.5	c.634	T		212	F	
FcgRIIIA‐158 F/V	for	NM_000569.6	NP_000560.5	c.634	T	TG	212	F	F/V
	rev	NM_000569.6	NP_000560.5	c.634	T	TG	212	F	F/V
FcgRIIIA‐158 V/V	for	NM_000569.6	NP_000560.5	c.634	T	G	212	F	V
	rev	NM_000569.6	NP_000560.5	c.634	T	G	212	F	V

### PCR amplification

PCR products were amplified in a 25 µL reaction volume containing 0.3 µmol/L of each primer, 1.5 µmol/L MgCl_2_, 200 µmol/L of each dNTP, 0.02 U µl^‐1^ DNA polymerase (kappa robust; kappa Biosystems, KK5024), and 25 ng DNA of the sample. PCR cycle conditions are given in Table 3 and 4.

**Table 3:** PCR primers and annealing temperatures used for the PCR amplification.

PCR	Primers	Sequence	Amplicon Length	Annealing Temperature
FCGR2A_Ex04	FCGR2A_Ex04_fw	GCATCTTCATTTCTGTCTGCCA	493	63 °C
	FCGR2A_Ex04_rev	CTCCAGTGCCCAATTTTGCT		
FCGR3A_Ex04	FGR3A_For03	GTGTTTACATTGAGTTCTCCTTC	873	63 °C
	FCGR3A_Ex04_rev	TCCTCCCAACTCAACTTCCC		

**Table 4:** PCR cycle sequencing conditions; 35 cycles.

PCR Step	Description	Temp (°C)	Duration (sec)
1	Initial Denaturation	95	180
2	Denaturation	95	20
3	Annealing	63	20
4	Elongation	72	30
5	Final Elongation	72	45

### Sanger sequencing

The PCR products were purified, and bi‐directionally Sanger sequenced at Microsynth (Balgach, Switzerland) using the two PCR primers for *FCGR2A*. In case of the *FCGR3A* gene an internal forward primer (*FCGR3A*_For02: GGGGTGTCTGTGTCTTTCAG) as well as the reverse PCR primer were used for the Sanger sequencing. The sequences were aligned against the reference sequences derived from GenBank (and any SNPs were recorded with Soft Genetics Mutation Surveyor v4.0.9 software).

### Statistical analysis

The distributions of positive versus negative clinical and radiological outcomes for patients in different genotypic groups were compared using the two‐sided Fisher’s exact test via Prism version 7.0a for Mac OSX (Graphpad Software, Inc) and corrected *p*‐values (Bonferroni correction) were calculated with the *p.adjust* function of the “stats” package (version 3.6.0) with R (http://www.R-project.org). A *p‐*value < .05 was considered statistically significant.

## Results

### Study population characteristics

Data were derived from 85 patients, of whom 49 (58%) were female. The mean age of patients at initial treatment with alemtuzumab was 35.8 years (SD 9.7). Patients received 12 mg of alemtuzumab administered intravenously daily for five consecutive days and again one year later for three consecutive days. The median expanded disability status scale (EDSS) at baseline was 2.8 (interquartile range [IQR] 2). Mean relapse frequency prior to treatment was 2.6 relapses in the last two years before commencement of alemtuzumab (SD 1.8). The mean number of treatments prior to alemtuzumab was 2.3 (SD 1.7) and included natalizumab (*n* = 28), fingolimod (*n* = 18), IFNβ (*n* = 9), dimethylfumarat (*n* = 6), glatirameracetat (*n* = 4), teriflunomide (*n* = 2), mitoxantrone (*n* = 1), azathioprine (*n* = 1), and siponimod (*n* = 1). Fifteen patients did not receive any pretreatment (Table 1).

### Clinical outcomes

The total number of patients who experienced at least one relapse during the first two years of the follow‐up period was 34 (40%). Twenty patients (24%) met the definition for confirmed disease progression (CDP). NEDA‐3, as defined by absence of clinical relapse, no confirmed EDSS disability progression, no new gadolinium enhancing lesions and no new or enlarging T2 lesions, was observed in 41 (49%) of patients. Severe IAR included according to the *Common Terminology Criteria for Adverse Events* (CTCAE), death, life‐threatening consequences, fetal damage, and need for urgent hospitalization and were observed in 30 patients (35%). A total of 24 (28%) patients developed SAD. Observed SAD entities included Graves’ disease (*n* = 16), idiopathic thrombocytopenic purpura (ITP) (*n* = 2), vitiligo (*n* = 2), autoimmune hepatitis (*n* = 1), autoimmune thyroid disease (AITD) (*n* = 5), and hemophagocytic syndrome (*n* = 1). Two patients developed more than one SAD (patient 1: Graves’ disease, ITP; patient 2: AITD, Vitiligo and autoimmune hepatitis). The mean time point of SAD manifestation was at 22 months (SD: 12.9, range: 6–51 months). Six patients (7%) developed acute acalculous cholecystitis (AAC).

### Prevalence of *FCGR3A* and *FCGR2A* genotypes

**Figure 1:**
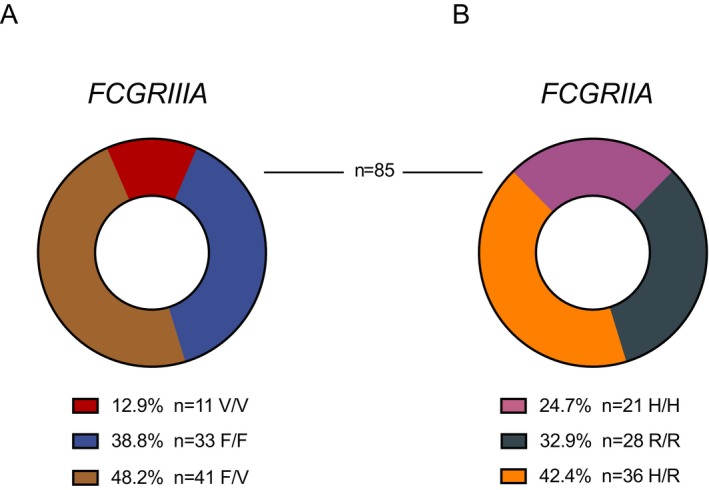
Fcγ receptor polymorphism frequency distribution for FcγRIIIA (A) and FcγRIIA (B) within the study cohort of 85 RR‐MS patients previously treated with alemtuzumab.

Prior to genotyping the patient samples via bi‐directional Sanger sequencing, PCR conditions were optimized and validated using six blood samples from individuals with known *FCGR3A* and *FCGR2A* genotypes. Among the 85 RR‐MS patients investigated, 11 (13%) carried the high‐affinity *FCGR3A* 158 V/V genotype, 41 (48%) were heterozygous carriers (*FCGR3A* 158 V/F), and 33 (39%) were homozygous for *FCGR3A* 158 F/F. With respect to the *FCGR2A* 131 polymorphism, 21 patients were (25%) genotyped as *FCGR2A* 131 H/H, 36 (42%) were heterozygous *FCGR2A* 131 R/H carriers, and 28 (33%) were homozygous for *FCGR2A* 131 R/R (Fig. 1). Frequency distribution of allelic variants in our study were similar to those reported for Caucasians in previous studies.^5^, ^6^

### Association of *FCGR* genotypes with clinical outcomes

**Figure 2:**
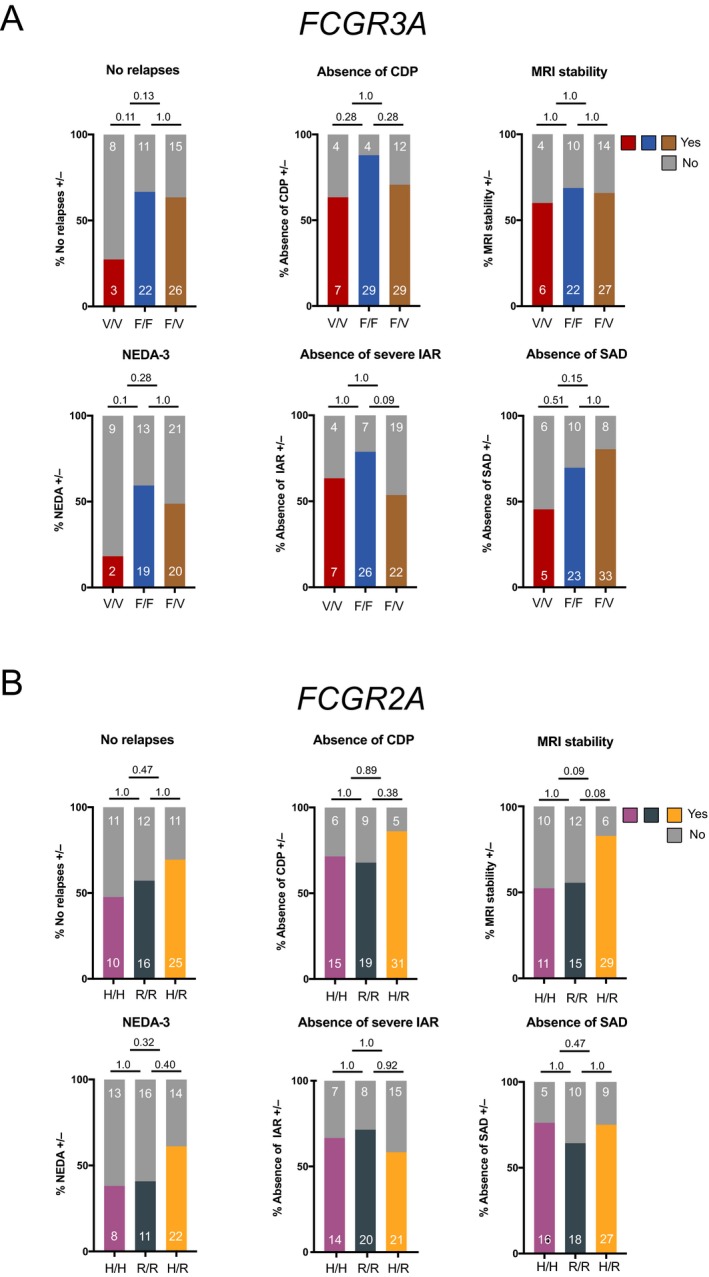
Clinical and radiological characteristics of RR‐MS patients after alemtuzumab therapy stratified by FcγR polymorphisms**. **(A) Data for homozygous 158V/V (high‐affinity), homozygous 158F/F and heterozygous 158F/V FcγRIIIA polymorphisms and (B) homozygous 131H/H (high‐affinity), homozygous 131R/R and heterozygous 131H/R FcγRIIA polymorphisms are depicted. For some characteristics not all patient data were available. For the following analyses the n was: 84, NEDA‐3/ FcγRIIIA (1 patient missing, F/F group); 83, MRI stability/ FcγRIIIA (2 patients missing, F/F and V/V groups); 84, NEDA‐3/ FcγRIIA (1 patient missing, R/R group) and 83, MRI stability/ FcγRIIA (2 patients missing, H/R and R/R groups). The remaining analyses were carried out on all 85 patients. Shown are percentages of the respective genotypic groups as bargraphs and absolute numbers of patients in white. Statistical analysis: Two‐sided Fischer’s exact test with Bonferroni correction for multiple testing was applied on absolute patient numbers. A *p‐*value < .05 was considered statistically significant. Abbreviations: CDP, confirmed disability progression; MRI, magnetic resonance imaging; NEDA, no evidence of disease activity; IAR, infusion‐associated reactions; SAD, secondary autoimmune disease

Twenty‐seven percent of patients with the *FCGR3A* 158 V/V genotype did not experience any relapses during the 2 year follow‐up period as opposed to 67% for *FCGR3A* 158 F/F and 63% for *FCGR3A* F/V carriers. Absence of CDP was documented for 63% of patients with V/V, 87 % of patients with F/F and 71% of patients with F/V genotypes. Sixty percent (V/V), 69% (F/F) and 66% (F/V) respectively showed MRI stability as defined by no new or enlarging T2 or gadolinium‐enhancing lesion. NEDA‐3 criteria were met by 18% of the V/V group versus 59% (F/F) and 49% (F/V). Severe infusion‐associated reactions were experienced by 36% of patients with V/V, 21% with F/F and 46% with F/V genotypes. In 55% of V/V secondary autoimmune diseases occurred as opposed to 30% of F/F and 20% of F/V (Fig. 2A).

**Figure 3:**
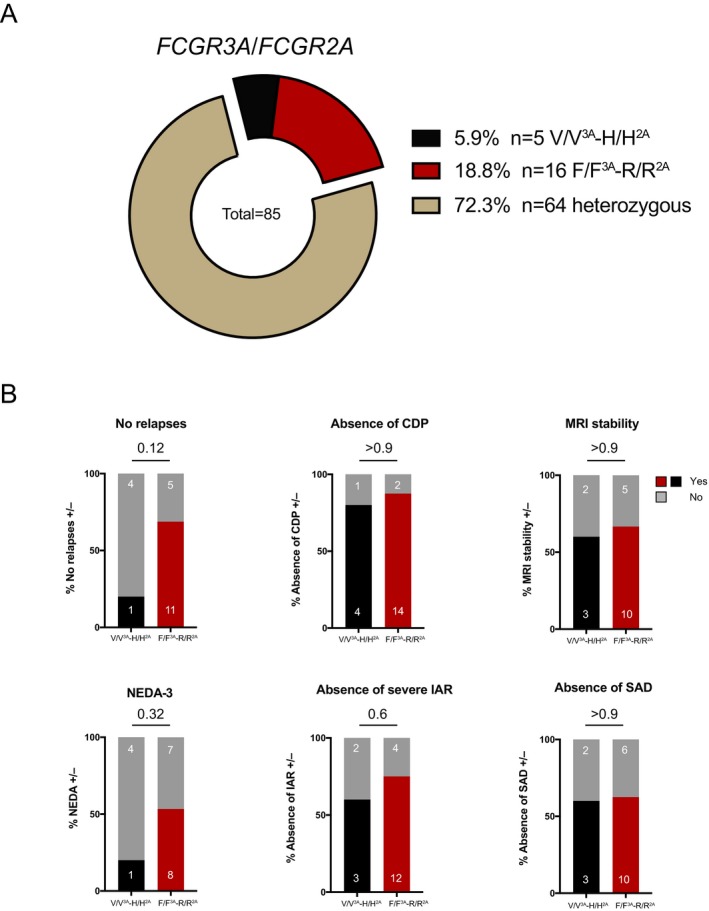
Stratification into double homozygous carriers (*FCGR3A* 158 V/V; *FCGR2A* 131 H/H, designated V/V^3A^‐H/H^2A^ and *FCGR2A* 158 F/F; *FCGR2A* 131 R/R, designated F/F^3A^‐R/R^2A^). (A) Fcγ receptor polymorphism frequency distribution for double homozygous carriers V/V^3A^‐H/H^2A^ and F/F^3A^‐R/R^2A^ within the study cohort of 85 RR‐MS patients previously treated with alemtuzumab. (B) Clinical and radiological characteristics of double homozygous RR‐MS patients after alemtuzumab therapy (n = 21) stratified by high‐ (V/V^3A^‐H/H^2A^) and low‐affinity (F/F^3A^‐R/R^2A^) FcγR polymorphisms. For some characteristics not all patient data was available. For the following analyses the n was: 20, NEDA‐3 (1 patient missing, F/F^3A^‐R/R^2A^ group); 20, MRI stability (1 patient missing, F/F^3A^‐R/R^2A^ group). The remaining analyses were carried out on all 21 patients. Shown are percentages of the respective genotypic groups as bargraphs and absolute numbers of patients in white. Statistical analysis: Two‐sided Fischer’s exact test was applied on absolute patient numbers. A *p‐*value < .05 was considered statistically significant. Abbreviations: CDP, confirmed disability progression; MRI, magnetic resonance imaging; NEDA, no evidence of disease activity; IAR, infusion‐associated reactions; SAD, secondary autoimmune disease.

Forty seven percent of the *FCGR2A* 131 H/H carriers were relapse‐free during the first 2 years after alemtuzumab treatment initiation compared to 57% of R/R and 69% of H/R carriers. Absence of CDP was noted in 71% (H/H), 68% (R/R) and 86% (H/R) of carriers, respectively. 52% (H/H), 56% (R/R) 83% (H/R) depicted stable MRI activity and NEDA‐3 status was achieved in 38% (H/H), 41% (H/R) and 61% (R/R) of study participants. Severe IAR occurrence was distributed as follows: 33% (H/H), 29% (R/R) and 42% (H/R). 24% (H/H), 36% (R/R), and 25% (H/R) developed SADs (Fig. 2B). Moreover, no statistically significant differences in clinical efficacy outcomes, the development of severe IARs and SADs were observed in patients homozygous for both high‐affinity variants (*FCGR3A* 158 V/V; *FCGR2A* 131 H/H, designated V/V^3A^‐H/H^2A^) as compared to patients homozygous for both low‐affinity FcγRs (*FCGR3A* 158 F/F; *FCGR2A* 131 R/R, designated F/F^3A^‐R/R^2A^) (Fig. 3). Altogether, *FCGR3A* and *FCGR2A* genotypes were not significantly associated with any of the aforementioned clinical or radiological outcomes following alemtuzumab therapy.

## Discussion

Our report is the first investigation into the impact of FcγR polymorphisms on clinical outcomes to lymphocyte‐depleting antibody therapy in patients with MS. No differences in clinical efficacy parameters, the development of severe IARs and SADs in response to alemtuzumab therapy were observed in patients with RR‐MS based on *FCGR3A* or *FCGR2A* polymorphisms. These data indicate that *FCGR3A* and *FCGR2A* polymorphisms are not associated with clinical outcomes to alemtuzumab in MS.

Our study was motivated by epidemiological investigations that reported an association between the low affinity *FCGR3A* 158 F/F genotype in patients with non‐Hodgkin B‐cell lymphoma and a poor clinical response to rituximab, presumably due to incomplete ADCC‐mediated depletion of tumor cells.^5^, ^6^, ^7^, ^14^ *In vitro* studies demonstrated that alemtuzumab depletes human lymphocytes through both Fc‐mediated ADCC and CDC.^4^ *In vivo*, cell depletion by alemtuzumab is reported to be predominantly mediated by ADCC and largely independent of complement as reported in transgenic mice expressing human CD52 15 and in an experimental murine model of T cell leukemia.^16^ While the mechanisms by which alemtuzumab depletes lymphocytes in humans and exerts its beneficial effects in MS are incompletely understood and might require complement‐dependent pathways, our data should not be interpreted to minimize the impact of ADCC as an important mechanism of action. Rather, FcγR polymorphisms may not strongly influence the clinical efficacy of alemtuzumab therapy. Our findings are based on a relatively small number of subjects (*n* = 85) and clearly require validation in larger cohorts. Moreover, we cannot exclude potentially confounding effects of neutralizing antibodies to alemtuzumab which may occur in individual patients within the first 2 years after treatment initiation.^17^

Given the clinical efficacy and increasing use of lymphocyte‐depleting antibodies in the therapeutic landscape of MS and other neurological diseases, our initial assessment provides incentive to conduct larger prospective studies in order to evaluate the impact of FcγR polymorphisms on clinical outcomes and *in vivo* mechanisms of therapeutic antibodies in MS.

## Author contributions

Christian W. Keller: Study concept and design, acquisition of data, analysis and interpretation, writing of the manuscript. Tobias Ruck: Study concept and design, acquisition of data, analysis and interpretation, critical revision of the manuscript for important intellectual content. Donal McHugh: Study concept and design, acquisition of data, analysis and interpretation, critical revision of the manuscript for important intellectual content. Steffen Pfeuffer: Acquisition of data, critical revision of the manuscript for important intellectual content. Catharina C. Gross: Acquisition of data, critical revision of the manuscript for important intellectual content. Catharina Korsukewitz: Acquisition of data, critical revision of the manuscript for important intellectual content. Nico Melzer: Acquisition of data, critical revision of the manuscript for important intellectual content. Luisa Klotz: Acquisition of data, critical revision of the manuscript for important intellectual content. Sven G. Meuth: Critical revision of the manuscript for important intellectual content. Christian Münz: Critical revision of the manuscript for important intellectual content. Falk Nimmerjahn: Critical revision of the manuscript for important intellectual content. Heinz Wiendl: Critical revision of the manuscript for important intellectual content. Jan D. Lünemann: Study concept and design, analysis and interpretation, writing of the manuscript, study supervision.

## Conflicts of Interest

C.W.K. received research support from the German Research Foundation (DFG) and the University of Zurich and travel expenses from Boehringer Ingelheim, the National Institute of Diabetes and Digestive and Kidney Diseases/NIH, and the National Institute on Aging/NIH. T.R. received travel expenses and financial research support from Genzyme and Novartis, and received honoraria for lecturing from Roche, Merck, Genzyme, Biogen, and Teva. S.P. received travel grants from Sanofi Genzyme and Merck Serono, lecturing honoraria from Sanofi Genzyme, Mylan Healthcare and Biogen and research support from Diamed and Merck Serono. C.C.G. received speaker honoraria, travel expenses for attending meetings, and research support from Biogen, Euroimmun, Genzyme, Mylan, Novartis, and Bayer Health Care. N. M. has received honoraria for lecturing and travel expenses for attending meetings from Biogen Idec, GlaxoSmith Kline, Teva, Novartis Pharma, Bayer Healthcare, Genzyme, Alexion Pharamceuticals, Fresenius Medical Care, Diamed, and BIAL, and has received financial research support from Euroimmun, Fresenius Medical Care, Diamed, Alexion Pharamceuticals, and Novartis Pharma. L.K. received compensation for serving on scientific advisory boards (Janssen, Novartis, Roche, Sanofi Genzyme); speaker honoraria and travel support (Biogen, Merck, Novartis, Roche, Sanofi Genzyme, Santhera); research support (Biogen, Merck, Novartis). S.G.M. received honoraria for lecturing, and travel expenses for attending meetings from Almirall, Amicus Therapeutics Germany, Bayer Health Care, Biogen, Celgene, Diamed, Genzyme, MedDay Pharmaceuticals, Merck Serono, Novartis, Novo Nordisk, ONO Pharma, Roche, Sanofi‐Aventis, Chugai Pharma, QuintilesIMS and Teva. His research is funded by Almirall, Amicus Therapeutics Germany, Biogen, Diamed, Fresenius Medical Care, Genzyme, Merck Serono, Novartis, ONO Pharma, Roche, and Teva. H.W. received honoraria for acting as a member of Scientific Advisory Boards for Biogen, Evgen, Genzyme, MedDay Pharmaceuticals, Merck Serono, Novartis, Roche Pharma AG, and Sanofi‐Aventis as well as speaker honoraria and travel support from Alexion, Biogen, Cognomed, F. Hoffmann‐La Roche Ltd., Gemeinnützige Hertie‐Stiftung, Merck Serono, Novartis, Roche Pharma AG, Genzyme, TEVA, and WebMD Global. He is acting as a paid consultant for Abbvie, Actelion, Biogen, IGES, Johnson & Johnson, Novartis, Roche, Sanofi‐Aventis, and the Swiss Multiple Sclerosis Society. J.D.L. received grant support, consultant and lecture fees from Novartis, Merck, Sanofi‐Genzyme, Bayer Pharma, Pfizer.
